# Effects of fine grinding on mid-infrared spectroscopic analysis of plant leaf nutrient content

**DOI:** 10.1038/s41598-023-33558-5

**Published:** 2023-04-18

**Authors:** Caleb R. Whatley, Nuwan K. Wijewardane, Raju Bheemanahalli, K. Raja Reddy, Yuzhen Lu

**Affiliations:** 1 Department of Agricultural and Biological Engineering, Mississippi State University, Mississippi State, MS 39762 USA; 2 Department of Plant and Soil Sciences, Mississippi State University, Mississippi State, MS 39762 USA; 3 Department of Biosystems and Agricultural Engineering, Michigan State University, East Lansing, MI 44824 USA

**Keywords:** Optical spectroscopy, Plant physiology, Infrared spectroscopy

## Abstract

Fourier transform mid infrared (FT-MIR) spectroscopy combined with modeling techniques has been studied as a useful tool for multivariate chemical analysis in agricultural research. A drawback of this method is the sample preparation requirement, in which samples must be dried and fine ground for accurate model calibrations. For research involving large sample sets, this may dramatically increase the time and cost of analysis. This study investigates the effect of fine grinding on model performance using leaf tissue from a variety of crop species. Dried leaf samples (N = 300) from various environmental conditions were obtained with data on 11 nutrients measured using chemical methods. The samples were scanned with attenuated total reflectance (ATR) and diffuse reflectance (DRIFT) FT-MIR techniques. Scanning was repeated after fine grinding for 2, 5, and 10 min. The spectra were analyzed for the 11 nutrients using partial least squares regression with a 75%/25% split for calibration and validation and repeated for 50 iterations. All analytes except for boron, iron, and zinc were well-modeled (average R^2^ > 0.7), with higher R^2^ values on ATR spectra. The 5 min level of fine grinding was found to be most optimal considering overall model performance and sample preparation time.

## Introduction

Fourier-transform infrared (FTIR) spectroscopy is a popular technique in agricultural research due to its flexibility in various applications, including but not limited to quality analysis for livestock and crop products^1–3^, phenotyping^4–6^, and characterization of physical and chemical properties for soil and biological material^7–10^. In particular, FTIR spectroscopy can be used as an alternative or complementary method to more traditional chemical procedures for multiple nutrient analysis in plants and soil, which is important for determining plant nutrient uptake and managing fertilizer application^11^. These traditional chemical analysis procedures for nutrient determination generate reliable chemical data but are costly, time-consuming, and destructive to the sample. By comparison, FTIR spectroscopy can be conducted quickly with ease and derive multiple traits using a single measurement, with the only actual cost being the fixed cost of a spectrometer system. An additional benefit is that FTIR reflectance spectroscopy does not destroy the sample during testing, preserving it for further use. Thus, FTIR is potentially superior in cases that do not require the degree of accuracy that traditional chemical analysis provides.

FTIR may refer to the near infrared range (approximately 12,500–4000 cm^−1^) or mid infrared range (approximately 4000–400 cm^−1^) of the electromagnetic spectrum. Fourier-transform near infrared (FT-NIR) has been widely used for soil and plant nutrient analysis due to the advantages it has in terms of how little sample preparation is needed, the low material cost, and the increased transparency of H_2_O in the NIR region compared to the mid infrared (MIR) region. However, absorption bands in the NIR region are mostly comprised of weak overtones of the fundamental absorption bands within the MIR region, which increase spectral complexity and therefore increase the computational complexity needed to extract useful information^12^. By comparison, functional group fundamental absorption bands typically occur in the MIR region, which are more robust and accessible to interpretation than the overtones in visible and near-infrared regions^13^.

There are multiple ways that Fourier-transform mid infrared (FT-MIR) spectroscopy can be conducted. Two methods are diffuse reflectance Fourier-transform (DRIFT) and attenuated total reflectance (ATR), both of which are used in this study. DRIFT spectroscopy directly measures the diffuse light reflected from the surface of a sample and has been extensively used for agricultural applications, particularly in the analysis of soils. DRIFT has been shown to be very effective for determining specific soil properties, such as organic and inorganic carbon, pH, total nitrogen, aluminum, and clay^7,14–16^. The ATR technique utilizes a highly refractive crystal (often a diamond due to its hardness) to repeatedly bounce an infrared (IR) beam within the crystal. The sample is pressed onto the surface of the crystal using a pressure arm, creating a contact point between the sample and the crystal at which the IR beam will reflect. The beam's interaction with the sample/crystal boundary creates an evanescent wave that penetrates slightly into the sample. The beam is attenuated by the absorption of radiation from the evanescent wave and then detected by the spectrometer. ATR soil studies have been able to predict specific characteristics such as nitrates, clay, and inorganic carbon with accuracy comparable to DRIFT^17,18^. While DRIFT is a more significant focus for FT-MIR spectroscopy research, ATR has certain advantages over DRIFT, such as reduced sample preparation time and the lack of specular artifacts^18^.

While FT-MIR spectroscopy often yields more accurate results than visible or near-infrared, its drawback is the sample preparation requirement^19–21^. For DRIFT, samples must be dried and finely ground to homogenize the sample and prevent specular reflections, which can obscure crucial spectral information. Guillou et al. demonstrated the benefit of fine-grinding soils for model performance^22^. They achieved higher prediction accuracy for clay, sand, and organic carbon with a particle diameter of 0.106 mm compared to particle diameters of ~ 2 mm. Several other studies have also showed that fine-grinding soil is a necessary step for the DRIFT technique^23–25^. The need for sample preparation is not as strict when using ATR considering the lack of specular effects. However, the quality of ATR spectra depends on the amount of contact with the ATR crystal; therefore, a smaller particle diameter will minimize the presence of microscopic air pockets and produce better results. As the measurement area is very small, fine grinding is also needed to promote sample homogeneity^18^. This sample preparation requirement presents the need for additional equipment, such as an oven and grinding mill, which can be very costly. The time dedicated to sample preparation is also an expensive factor in terms of time and human resources, especially in the case of model calibrations, where large sample sets are needed.

Many studies now exist on the use of FT-NIR spectroscopy for macro and micronutrient determination in plant leaves with varying levels of success in terms of accuracy^3,26–28^. Some studies have also compared model results between fresh leaf and powdered leaf spectra, with higher prediction accuracy achieved using powdered leaf^29,30^. However, while research on FT-MIR spectroscopy and plant material has recently expanded, there is still a limited application to nutrient prediction models. Studies that have compared NIR and MIR prediction models for leaves have achieved higher success on MIR spectra, furthering the case for the use of FT-MIR spectroscopy on plant material^11,31^. A study by Bekiaris et al. determined that ATR and DRIFT FT-MIR techniques are well-suited to plant material analysis regarding spectral quality, but did not apply this to predictive models^32^. To the best of our knowledge, only one other study exists on the multiple nutrient analysis of leaves using FT-MIR^33^. While fine-grinding recommendations for FT-MIR spectroscopy are documented for soils, no research exists presently on grinding recommendations for the use of leaves in FT-MIR spectroscopy. Due to the advantages of fine-grinding in soil research and the cost involved, there is a need to investigate the importance of fine-grinding on plant leaf FT-MIR spectroscopy and provide recommendations for standard laboratory scanning. This study aimed to provide firm conclusions on fine-grinding recommendations for laboratory protocols on plant leaf FT-MIR scanning. To this end, two main objectives were devised: (i) evaluate the effect of fine grinding on plant leaf model predictions and uncertainties of eleven different macro and micro nutrients: nitrogen (N), phosphorus (P), potassium (K), calcium (Ca), magnesium (Mg), manganese (Mn), iron (Fe), copper (Cu), boron (B), zinc (Zn), and sulfur (S) content, and (ii) compare grinding effects between ATR and DRIFT techniques.

## Results

### Particle diameter

**Figure 1:**
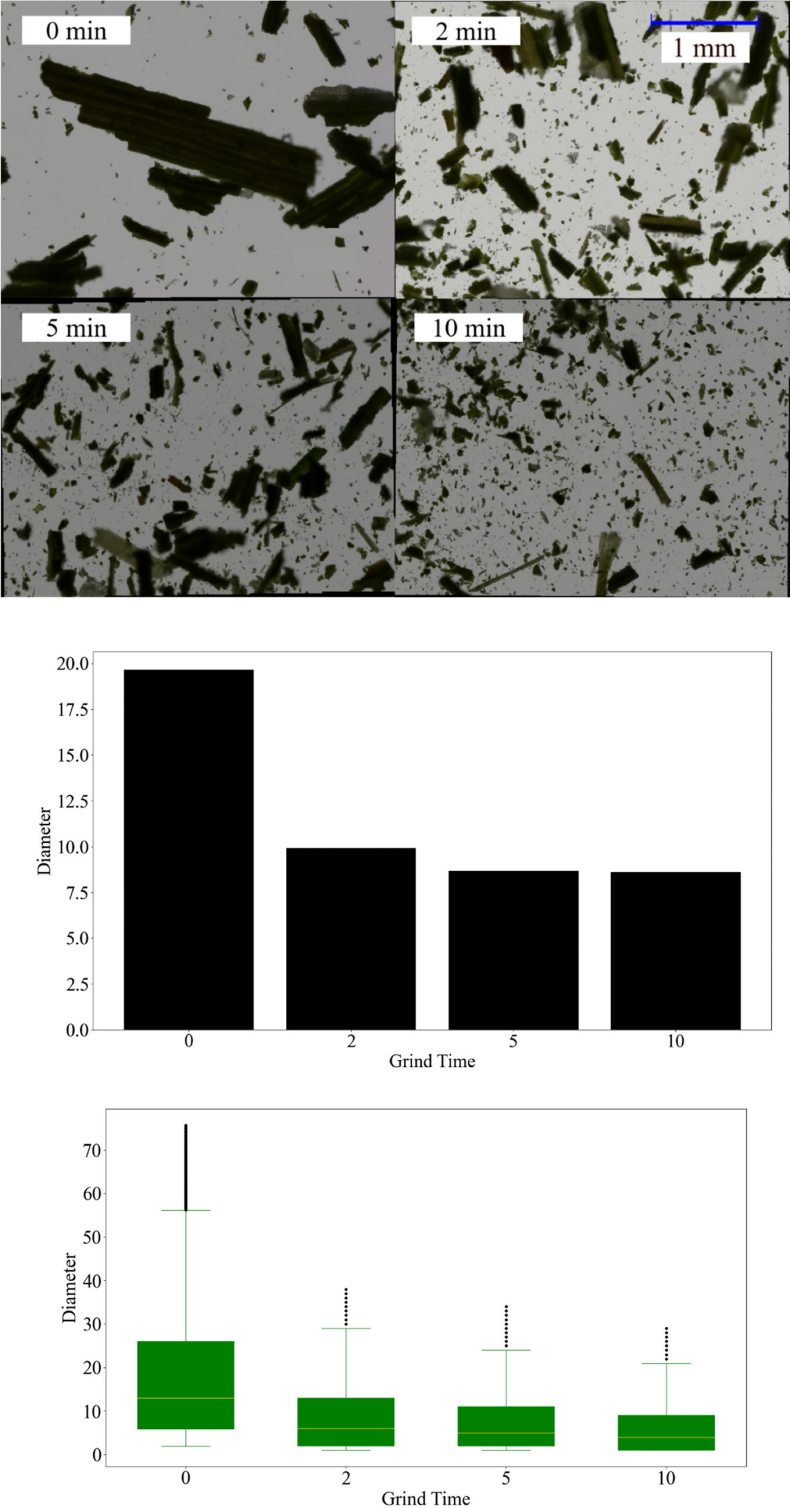
Top—Microscope images of dried rice leaf after 0, 2, 5, and 10 min of fine grinding. Middle—Average particle diameter (µm) of all plant species for each level of grinding (minutes). Bottom—Distribution of particle size for each level of fine grinding. Outliers are indicated by the black points.

Figure 1 shows the visual reduction in particle size for a leaf sample with each level of fine grinding. Before fine grinding was performed, the average particle diameter for all analyzed samples was 19.64 µm, with a standard deviation of 18.07 µm. The plant leaf with the largest average particle diameter was mustard, with an average particle diameter of 24.82 µm and a standard deviation of 17.42 µm. The lowest was soybean, with an average particle diameter of 16.2 µm and a standard deviation of 11.56 µm (see supplementary table S1). After fine grinding for 2 min, the average particle diameter for all samples decreased by 47.5%. Fine grinding to 10 min total resulted in a further decrease of 9.7% from the 2-min level. The decline in particle diameter change, as shown in Fig. 1, is possibly due to the limitations of the mill for grinding dried plant matter. Skewness remained above 1 for all grinding levels, indicating that particle diameter distribution was heavily right-skewed. A low kurtosis for each grinding group suggests that few outliers are present relative to the overall particle diameter distribution. This is expected, as outliers were screened and removed before statistical analysis.

### Spectral differences

**Figure 2:**
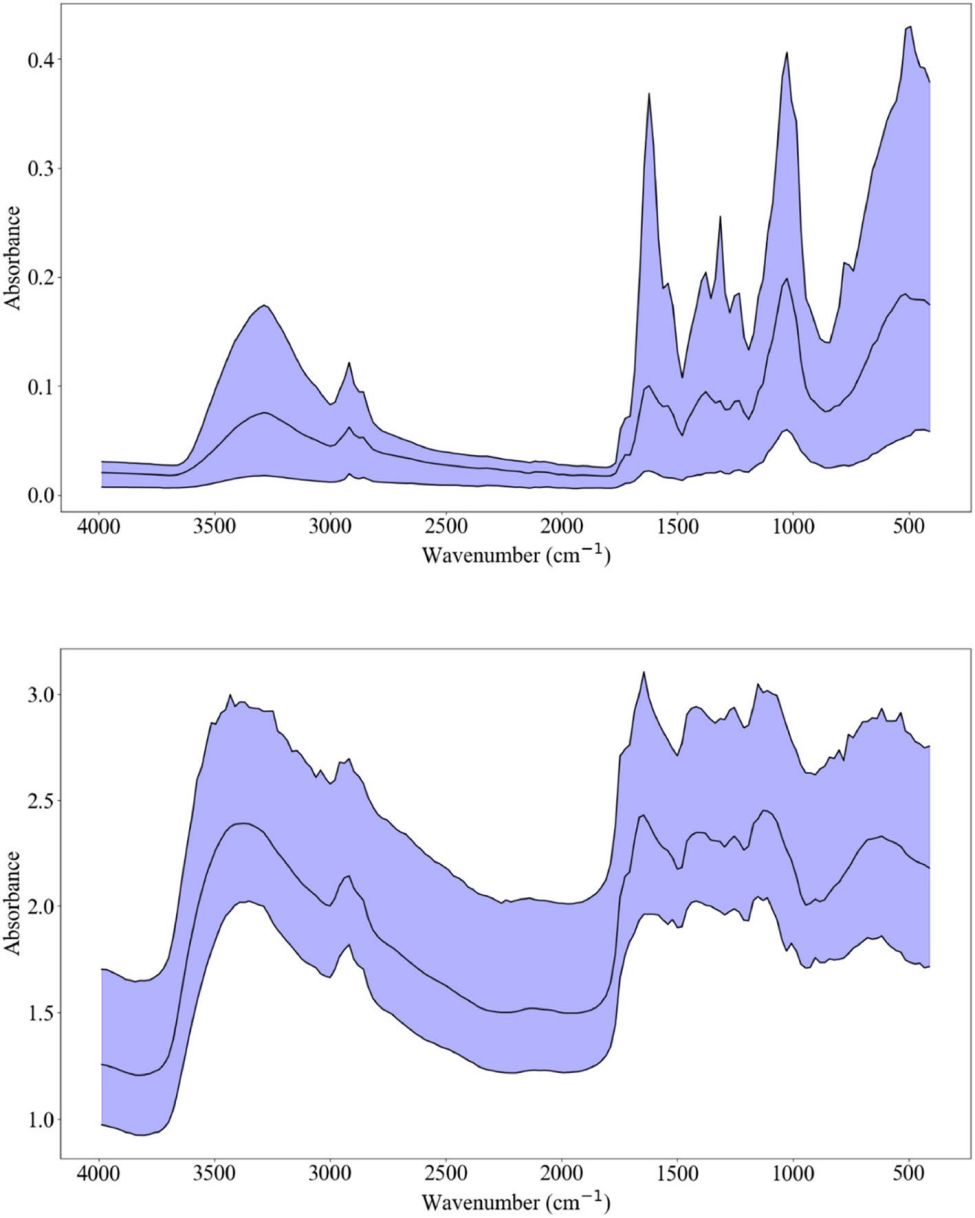
Minimum, mean, and maximum spectra of all samples for ATR (top) and DRIFT (bottom). Wavenumber is measured in cm^−1^.

The leaf spectra contained peaks and troughs that were primarily identical between ATR and DRIFT (Fig. 2). Defined absorbance peaks were located around 1600 and 1100 cm^−1^ and broad peaks were centered around 3400 and 600 cm^−1^. Absorption in these regions is consistent with carbon–nitrogen (C–N) and hydrogen–nitrogen (N–H) bonds. For both ATR and DRIFT, several absorbance peaks were observed between 1600 and 1000 cm^−1^. This is within the fingerprint region of the mid infrared spectrum and may therefore be an indicator of various bond types^34^. Low absorbance occurred between 2800 and 1700 cm^−1^ and beyond 3500 cm^−1^ for ATR. Sharper peaks and lower signal strength characterized ATR spectra compared to DRIFT spectra which had softer peaks and relatively high signal strength. The lower signal strength in ATR spectra may be attributed to poor radiation penetration, which has been observed in ATR spectra of dried and ground soils^35^.

**Figure 3:**
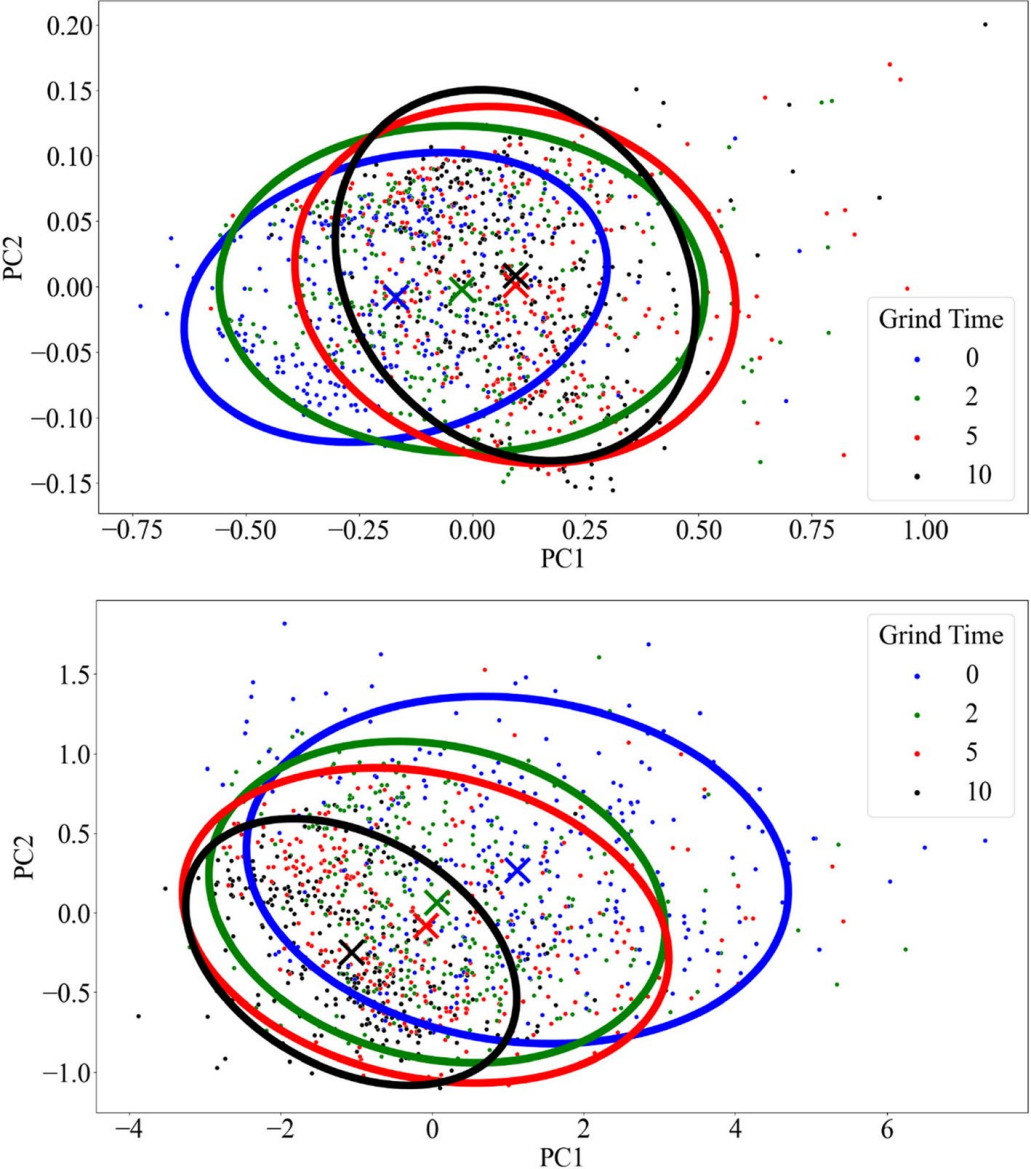
95% confidence ellipses of sample spectral data for ATR (top) and DRIFT (bottom) spectroscopy in PC space for different levels of grinding (minutes). Measures of the center for each confidence ellipse are indicated by the ‘x’ corresponding to the category color.

Figure 3 shows the variation of sample spectra with fine grinding in PC space as 95% confidence ellipses which contained 95% of data points at each grinding level. These PCs accounted for 96% of the total variance. Results from Hotelling’s T^2^ test showed that pc score centers for all grinding levels were significantly different from each other (*p* < 0.05), with the exception of the 5- and 10-min grinding levels for ATR spectra. For DRIFT spectra, variation in spectral distribution decreased visibly with each reduction in particle size, with a significant decrease in ellipse area between 0 and 10 min of fine grinding. This is attributed to the reduction in spectral variation with fine grinding, as shown by Guillou et al. for soil samples^22^. The ellipses also showed a trend of left and downward shifting for each level of fine grinding, indicating that fine grinding has a definite effect on spectra. The opposite direction was apparent for ATR spectra, as each level of grinding resulted in a right and slightly upward shift of ellipses. The exception was the change between 5 and 10 min of fine grinding, as the measures of the center are nearly identical. While the ellipse shape changed markedly between 0 and 10 min of fine grinding, the shape between 5 and 10 min of fine grinding is relatively similar, indicating similar spectral variations, which aligns with the results from Hotelling’s test.

### Model results

Supplementary tables S2 and S3 summarize the performance metrics of the models. Out of the 11 properties measured, only 8 were modeled well enough to achieve R^2^ values of 0.7 or greater. Model results for Fe, B, and Zn were not considered for further analysis due to R^2^ values being consistently below 0.6. It was noted that the R^2^ values for individual properties varied considerably with respect to each other at each fine grinding level. Nitrogen was the best-predicted property using ATR and DRIFT, with a mean R^2^ of 0.94 and 0.93, respectively, for all fine grinding levels. Manganese was the lowest performing property (out of the 8 kept properties) for ATR and DRIFT, with a mean R^2^ of 0.76 and 0.68, respectively. Predictions on ATR spectra were overall more accurate than DRIFT spectra. This may be due to the lack of specular artifacts in ATR spectra that can occur using reflectance methods^18^. Another possibility is the lack of atmospheric effects, leading to sharper and more defined absorbance peaks.

**Figure 4:**
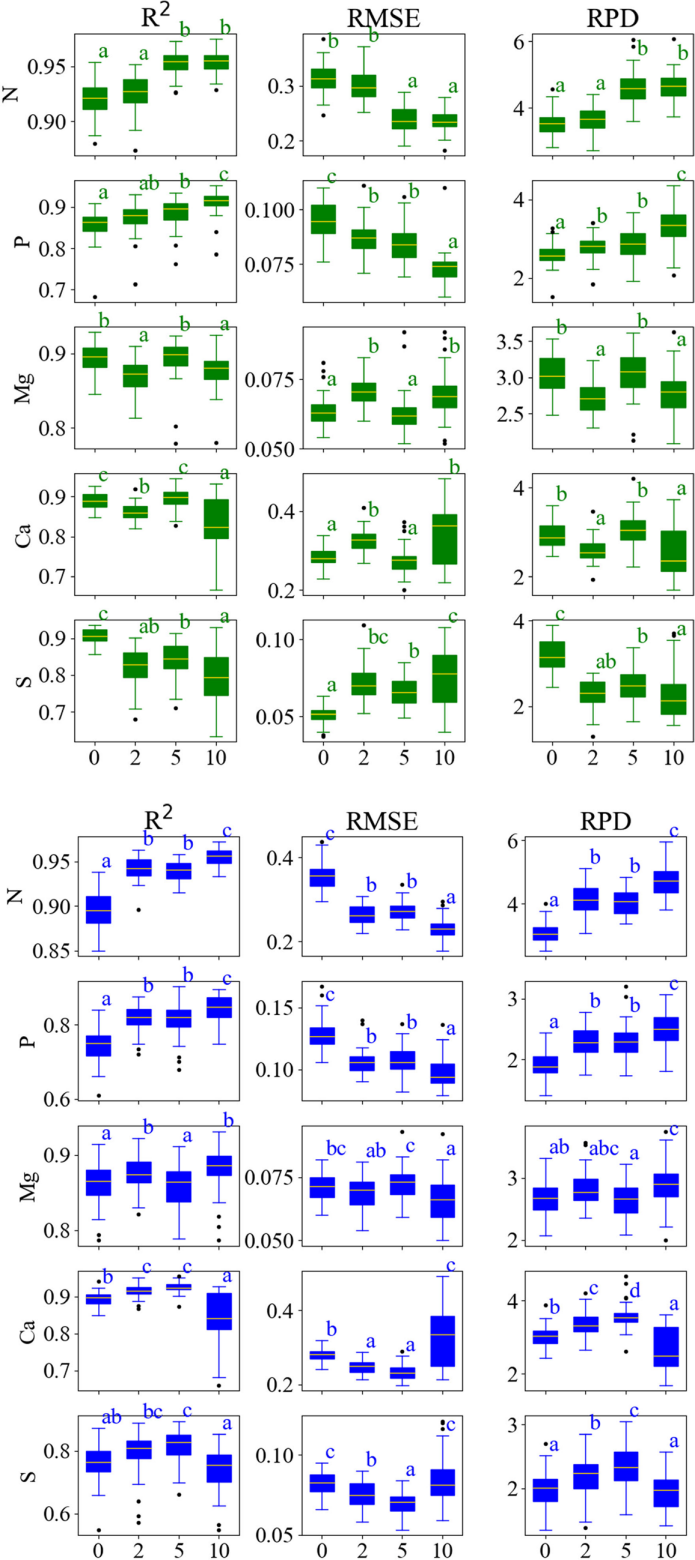
Boxplots of R^2^, root mean square error (RMSE), the ratio of performance to deviation (RPD), and bias for the prediction of 4 properties for each level of fine grinding for ATR (top) and DRIFT (bottom) spectroscopy techniques. Each boxplot is labeled with Tukey’s significance letters (*p* < 0.05) for comparison purposes. Fine grinding levels are in minutes.

The effect of fine grinding on model predictions largely depended on the predicted property (Fig. 4). Nitrogen and P R^2^, RMSE, and RPD values improved with each grinding level. The significance of these improvements differed between ATR and DRIFT. The improvement between 2 and 5 min of fine grinding was found to be statistically significant (*p* < 0.05), but not between 0 and 2 min or 5 and 10 min. The opposite was the case with DRIFT, as the improvement between 0 and 2 min and 5 and 10 min was statistically significant, but not the improvement between 2 and 5 min. Prediction accuracy of Mg decreased between 0 and 2 min, increased between 2 and 5 min, and decreased again between 5 and 10 min for ATR. Once again, the opposite trend was seen for DRIFT, as 2 and 10 min of fine grinding resulted in the most accurate predictions. Prediction accuracy for K did not change for either ATR or DRIFT between levels of fine grinding. Calcium predictions performed best at 0 and 5 min for ATR and 2 and 5 min for DRIFT. For Mn, little statistical change was observed in R^2^ values for each fine grinding level; however, RMSE values decreased with each fine grinding level for ATR and DRIFT (except the 2-min level in ATR spectra, as RMSE values increased). Manganese predictions also resulted in an unusually high range of bias values (-26.17 to 38.76) and RMSE values (64.12 to 152.12), which may be due to high variation in property measurements. For each fine grinding level, Cu R^2^ values for ATR improved, although RMSE values only approved significantly at 10 min. For DRIFT, Cu R^2^, RMSE, and RPD values were best for 0 and 10 min and the poorest for 5 min. Sulfur prediction accuracy experienced a downward trend for each fine grinding level for ATR, with 0 min being the most accurate and 10 min resulting in the lowest accuracy. Prediction accuracy for S for DRIFT peaked at 5 min and then decreased to its lowest point at 10 min.

For those R^2^, RMSE, and RPD values that changed significantly between fine grinding levels, the average percent change for each property is shown in Table 1. Changes to R^2^ were generally smaller compared to changes to RMSE and RPD. For ATR results, the most desirable changes were observed between 2 and 5 min of fine grinding, as two properties had improved R^2^ values, and four had improved RMSE and RPD values. No properties improved with 2 min of fine grinding for R^2^, and only phosphorus improved in RMSE and RPD. The 10-min grinding level resulted in a mix of improvements and deteriorations for all statistics.

**Table 1:** Average percent change in R^2^, root mean square error (RMSE) and the ratio of performance to deviation (RPD) for 8 measured chemical properties for ATR (top) and DRIFT (bottom) techniques between different levels of fine grinding.

Property	% Change in R^2^			% Change in RMSE			% Change in RPD		
	2	5	10	2	5	10	2	5	10
N (%)	NS	2.89%	NS	NS	− 20.63%	NS	NS	26.11%	NS
P (%)	NS	NS	3.02%	− 7.60%	NS	− 13.25%	7.71%	NS	15.43%
K (%)	NS	NS	NS	NS	NS	NS	NS	NS	NS
Ca (%)	− 3.17%	4.23%	− 6.78%	14.01%	− 15.17%	23.62%	− 12.16%	18.57%	− 16.28%
Mg (%)	NS	NS	NS	11.18%	− 10.31%	10.04%	− 10.46%	12.15%	− 8.90%
Mn (ppm)	NS	NS	NS	11.90%	− 17.02%	− 9.02%	− 10.87%	20.72%	9.83%
Cu (ppm)	NS	NS	3.96%	NS	NS	− 7.29%	NS	NS	7.15%
S (%)	− 9.44%	NS	− 5.42%	40.81%	NS	13.87%	− 28.44%	NS	− 9.49%

Property	% Change in R^2^			% Change in RMSE			% Change in RPD		
	2	5	10	2	5	10	2	5	10
N (%)	5.14%	NS	1.74%	− 25.17%	NS	− 14.44%	34.96%	NS	16.97%
P (%)	9.84%	NS	3.90%	− 17.13%	NS	-9.08%	19.76%	NS	9.60%
K (%)	NS	NS	NS	NS	NS	NS	NS	NS	NS
Ca (%)	2.33%	NS	− 8.31%	− 11.23%	NS	39.30%	11.67%	6.40%	− 25.02%
Mg (%)	1.65%	− 1.67%	2.40%	NS	5.71%	− 7.76%	NS	NS	9.52%
Mn (ppm)	23.22%	NS	NS	− 18.35%	NS	− 8.06%	20.63%	NS	NS
Cu (ppm)	NS	NS	NS	NS	NS	NS	NS	NS	NS
S (%)	NS	NS	− 9.29%	NS	NS	NS	NS	NS	NS

A positive change in R^2^ or RPD corresponds to an improvement in model accuracy, while a negative change is desirable for RMSE. Columns 2, 5, and 10 refer to fine grinding levels in minutes. For example, a value in column ‘2’ refers to the percent change between no fine grinding and two minutes of fine grinding. A negative value means the statistic value decreased from the previous grinding level. A value of ‘NS’ means that the percent change between fine grinding groups was not statistically significant at *p* < 0.05 compared to no fine grinding.

Similar to ATR results, the 10-min level resulted in improvements and deteriorations. For DRIFT, the only improvement at 5 min was RPD for Ca. Magnesium R^2^ and RMSE values decreased and increased, respectively, and all other changes at 5 min were insignificant. The majority of desirable changes were observed with 2 min of fine grinding, as five properties had improved R^2^ values, and four had improved RMSE and RPD values.

## Discussion

The average particle diameter for all samples did not decrease much despite fine grinding for 10 min. Grinding for 2 min reduced the average particle diameter by half, but further grinding produced almost negligible results. The coarse grinding step was mostly sufficient for reducing particle diameter, given the average particle diameter of ~ 20 µm. This is likely due to the softness of leaf particles compared to the complex mineral structures of soil particles which require fine grinding to reduce particle diameter effectively. However, fine grinding had a definite effect on model performance, possibly due to additional homogenization and the breakup of leaf and cell structures, exposing more chemical bonds.

Determining an accurate particle diameter distribution encompassing all plant types proved difficult due to the physical properties of leaf particles. Measurements for particle diameter differed between the plant types used, as there were differences in leaf hardness and structure, such as additional veins and fiber content in some plant species. For instance, the particle diameter of soybean leaf, which is relatively soft, was reduced to an average of 6.52 µm after 10 min of fine grinding, while triticale average particle diameter was decreased only to 11.17 µm. A more significant issue is the increase in average particle diameter occasionally after fine grinding, particularly after 10 min. An example is cereal rye, for which the average particle diameter increased from 10.75 to 14.25 µm between 5 and 10 min of fine grinding. We hypothesize that this is due to the aggregation of very fine particles, which could result from electrostatic attraction or the physical shape of particles themselves, forming a cohesive effect. The microscope software was unable to differentiate individual particles if significant overlap occurred. This direct image analysis method was the only feasible option for particle diameter measurement due to limitations in equipment availability. Other techniques, such as laser or X-ray diffraction, are not applicable in this circumstance due to leaf particles' varied and amorphous nature. Despite the limitations, these results were sufficient to provide a reasonably accurate estimate of leaf particle diameter distribution.

In all cases except for DRIFT models of Mn, changes to R^2^ were relatively small, less than 10% between levels of fine grinding. Changes to RMSE and RPD were more significant overall, often exceeding 10%. Therefore, fine grinding is particularly effective at reducing prediction errors but not so helpful at improving model R^2^ for properties that will enhance with fine grinding. This error reduction aligns with another study that showed a decrease in RMSE and RMSE variation in soil models after fine grinding^25^.

The noticeable improvements in DRIFT models after 2 min and 10 min of fine grinding, as seen by the decrease in variation in Fig. 3 and the improvements to R^2^, RMSE, and RPD in Table 1, indicate that fine grinding had a definite effect, which may be the reduction of specular reflections due to the breakdown of larger particles as well as homogenization^36^. Fine grinding does not appear to have as much effect on models of ATR spectra, although RMSE did improve for most properties after 5 min of fine grinding. This could be attributed to sample/crystal contact being essential for quality ATR spectra. Because leaf particles are more pliable than soil particles, the pressure arm's force exerted on the sample may be sufficient for ensuring good sample/crystal contact regardless of particle size. These results suggest that ATR may be a better alternative to DRIFT for the analysis of leaf samples, as ATR spectra produced better models on average and are less dependent on particle size than DRIFT. Unlike DRIFT, ATR may also be used to scan intact leaves which would remove the sample preparation step. This method has been proven to be useful in certain applications, such as the analysis of fresh leaves to monitor environmental pollution^37^. However, further research needs to be performed to determine the viability of intact leaves for nutrient model calibrations. Finally, the ease of use for ATR spectroscopy and the lack of need for a separate reference improves the case for ATR methods over DRIFT, especially when scanning large numbers of samples.

While fine grinding generally improved model performance, results were not equal among the various properties. Nitrogen and P were the only properties that showed better model performance with each level of fine grinding. Potassium predictions were not affected at all by fine grinding for both ATR and DRIFT. Excessive grinding degrades model performance for some properties, especially Ca and S. A possible reason for this is the breakdown of chemical bonds resulting from grinding, as was suggested when fine grinding soils by Harada et al.^38^. Therefore, fine grinding should likely be avoided for studies seeking to analyze K, S, or Ca content in leaves.

The overall model performance for most analytes ranged from acceptable to very good. Nitrogen was the best predicted, which is expected as nitrogen bonds directly absorb energy in the MIR region. Other analytes were predicted based on their associated functional groups, which absorb in the MIR region rather than the analyte itself^39^. This indirect spectral representation is typical for most properties, as it has been shown in soil studies that various physical, chemical, and biological properties can be predicted from FT-MIR spectra^40^. Iron, B, and Zn predictions were comparatively poor, especially for DRIFT spectra. These nutrients are not typical targets of FT-MIR spectroscopic analysis as they are not well-represented in the mid infrared region. One soil study did achieve good Fe predictions with an R^2^ of 0.88, but Zn predictions were extremely poor^21^. A survey of MIR analysis of nutrient properties in tree foliage achieved an R^2^ of 0.79 for Fe predictions^33^, comparable to our findings on Fe predictions for ATR spectra at the 10-min level of fine grinding (R^2^ = 0.77). While there may be potential for the use of FT-MIR spectroscopy to predict Fe content in leaves, B and Zn are not ideal analytes for this method.

Deciding on the fine grinding needed for FT-MIR analysis of leaf material should consider several factors. As stated, not all models of properties responded positively to fine grinding; however, the degree of model improvement or decline should be considered. For example, while prediction accuracy on DRIFT spectra of calcium content reached the lowest point at 10 min of fine grinding, the average and highest R^2^ values for that level were 0.85 and 0.93, respectively, which is still a good model and is comparable to Ca prediction results from a study using mid infrared spectra of conifer foliage^31^. Conversely, N predictions on DRIFT spectra improved with each level of fine grinding. However, the R^2^ value for predictions on non-fine ground samples was 0.9, which could still be considered an excellent model, although RMSE did decrease substantially after 10 min of fine grinding. Therefore, the need for fine grinding may depend on the degree of accuracy desired for model predictions and the properties involved. For a study analyzing one or a few properties, the need for fine grinding could be adjusted based on the model performance for those analytes. If a study seeks to explore a variety of properties, such as in this one, a compromise on fine grinding time should be made to maximize the effectiveness of models. According to the results of this study, the 5-min level of grinding produced the most significant benefit to overall model performance for ATR. While the model performance of DRIFT spectra was slightly better at 10 min, there was virtually no decline in model performance at 5 min of fine grinding.

Another critical factor to consider is the time and cost dedicated to sample preparation due to fine grinding, as Guillou et al. concluded in a study on grinding recommendations for soils^22^. Increasing the amount of fine grinding time can dramatically increase sample preparation time with large numbers of samples. For a set of 300 samples, using a mill that can grind 2 samples at once, fine grinding for 2 min would increase preparation time by 5 h. Fine grinding for 10 min with the same conditions would increase preparation time by 25 h. This preparation time is separate from the other tasks that are needed for fine grinding, such as loading and unloading samples from the mill and cleaning and drying the milling cups. While mills are available that allow for multiple samples to be ground at once, such an acquisition would significantly increase initial costs. This time spent may also incur monetary charges if workers are being paid to process samples or indirect costs as sample processing time detracts from a researcher’s time that could be spent on other tasks. Wijewardane et al. discussed a cost estimate for soil grinding and stated that fine grinding added approximately $1 per sample, excluding equipment costs^25^. Given these time considerations and the model results of this study, we recommend 5 min of fine grinding as adequate for studies seeking to observe multiple analytes and maximize model performance.

## Methodology

### Samples

The leaf samples collected from different experiments were dried in a forced drier at 70 °C until a constant weight was reached. In brief, 12 plant types (Table 2) were grown under different climatic (UV-B, low and high temperatures, elevated CO_2_) or soil variables (low nutrient, water logging, salt, and drought) using Soil Plant Atmosphere Research (SPAR) chambers^41–44^, outdoor pot culture^45^, and field conditions following the appropriate institutional guidelines. Leaf samples collected across experiments were coarsely ground using a tumbler grinding method. All the samples were analyzed for 11 different chemical properties: N, P, K, Ca, Mg, Mn, Fe, Cu, B, Zn, and S content in a soil testing laboratory at Mississippi State University (http://extension.msstate.edu/agriculture/soils/soil-testing). The N content was measured by placing 0.25 g of dried leaf into an organic elemental analyzer (Vario Max, Elementar, Langenselbold, Germany). To measure all other chemical properties, 0.5 g of dried leaf was placed into a furnace for 4 h at 500ºC. It was then combined with 40 mL of dilute hydrochloric acid and analyzed using inductively coupled plasma optical emission spectrometry (Spectro Blue ICP-OES, AMETEK Inc, Pennsylvania, USA). See table S4 for detailed property statistics.

**Table 2:** Plant species used in this study.

Plant	Number of samples
Amaranth	30
Cereal rye	2
Corn	78
Cotton	30
Crimson clover	2
Mustard	2
Rice	15
Sesame	22
Sorghum	30
Soybean	55
Triticale	2
Wheat	32
Total	300

### Sample preparation and grinding

Approximately 5 cm^3^ of each leaf sample was placed into a labeled glass scintillation vial. Each sample was finely ground using a mill (MM 500/ MM 200, Retsch GmbH, Germany).This mill consists of stainless-steel milling cups which are used to grind the sample with 5 steel balls. After filling the cups with the sample and 5 stainless steel balls, the cups were firmly inserted into the grinder, which was then shaken at 25 Hz for a specified time. Ground samples were transferred back to their respective vials using a funnel. The milling cups and steel balls used for grinding were then cleaned using a dish soap and water mixture, rinsed with distilled water, and thoroughly dried to prevent cross-contamination. Using this method, up to 8 samples could be ground at one time. The final particle diameters of the ground samples were decided on the grinding time since the shaker frequency was set at a constant value (25 Hz). The leaf samples were first fine ground for 2 min to obtain different particle diameters. For the next particle diameter, samples were ground for an additional 3 min to account for 5 min of total grinding time. This process was continued to obtain a series of grinding times (i.e., different particle diameters): 2, 5, and 10 min. The samples were stored in a dry, room-temperature location among grinding steps.

### Particle size analysis

An optical microscope (VHX7000, Keyence, Osaka, Japan) and its accompanying software were used to measure particle diameter distributions. A set of 20 random samples was chosen to measure particle diameter distribution at each level of fine grinding (0, 2, 5, and 10 min of fine grinding), which included 9 plant types. Approximately 0.1 g of fine ground leaf was placed onto the microscope stage, which consisted of a glass plate illuminated from underneath. A microscope slide spread the sample across the stage to minimize particle overlap. The microscope software was used to capture and stitch images of the sample into one larger image to include all the particles on the stage. The magnification used varied between 200 × and 400 × depending on the size of the particles observed. Using a particle detection function of the software, individual particles were automatically recognized and measured. The software generated a spreadsheet containing multiple measurements for each particle, such as maximum diameter, area, and perimeter.

To avoid bias due to overlapping particles, the results were accepted only if the number of particles detected exceeded 1000; otherwise, the stage was cleaned, the sample reapplied, and imaged. As a few extremely large particles or image artifacts noticeably skewed the results, outliers were screened based on maximum diameter. To identify outliers in each image, the first quartile (Q1: the lowest 25%) and third quartile (Q3: the lowest 75%) of the particles were calculated to determine the interquartile range (IQR). A particle was declared an outlier and removed from the data set if its maximum diameter was greater than Q3 + 1.5*IQR, which is a common method for declaring outliers based on the interquartile range. The average particle diameter was then calculated for each plant species, the overall sample set, and each level of fine grinding.

### Spectral acquisition

Spectral data were collected from each sample for each grinding level using an FT-MIR spectrometer using ATR and DRIFT techniques in the form of interchangeable modules designed for each technique (Alpha II, Bruker, Massachusetts, USA). The wavenumber of each spectrum ranged from 4000 to 400 cm^−1^. Each spectrum was obtained as an average of 32 instantaneous scans using the OPUS software (Alpha II, Bruker, Massachusetts, USA), which was used to control and acquire spectra from the spectrometer. To collect DRIFT spectra, the module designed for DRIFT spectroscopy was attached to the spectrometer. The module consisted of a chamber housing a slidable arm, which could hold a gold reference or sample cup 1 cm in diameter, both specifically designed to be used with that module. The arm could slide out of the chamber to load the reference or sample, then be reinserted to begin measurement. Using rubber-tipped forceps, the gold reference was carefully inserted into the spectrometer to measure a baseline. This was repeated before each sample was scanned. Once a baseline had been measured, a small metal scoop was used to load the ground leaf sample into the sample cup. The excess leaf was scraped from the cup using the scoop so that the surface of the ground leaf would be flush with the cup rim. This resulted in approximately 0.05 g of dried leaf being used per measurement. Using the forceps, the gold reference was replaced with the prepared sample cup, and a measurement was taken. After completion, the sample was returned to its vial. Residual particles were removed from the sample cup using blasts of air from a handheld air pump, and the scoop used to obtain the sample was wiped clean using a dry lab wipe. The air pump cleaned the module chamber to prevent buildup inside periodically.

As with DRIFT spectral acquisition, ATR spectra were collected using a module designed for ATR spectroscopy. The module consisted of a circular metal plate 8 cm in diameter, with a monolithic diamond 2 mm in diameter in the center. An adjustable pressure arm was located over the diamond, which acts as the measurement location. No particular reference was needed to measure the baseline; it was only necessary to ensure that the diamond was clean with no sample present to collect a baseline measurement. After measuring a baseline, a small amount of ground leaf was placed onto the diamond using a wooden stir stick. Approximately 0.03 g was used to cover the diamond fully. The pressure arm was lowered onto the sample, compressing it against the crystal, and a measurement was taken. The sample was placed back into the vial after measurement using the stir stick, and a funnel was placed into the vial. The module stage, crystal, and pressure arm were cleaned with wipes containing 75% alcohol, then dried using lab wipes. The funnel was cleaned using the air pump, and the wooden stir stick was replaced before the following sample. All DRIFT and ATR spectra were saved in a file format unique to the OPUS software but later converted to text files using a macro to perform the data analysis.

### Data analysis

The analysis was performed in Python (version 3.9, Python Software Foundation) using the libraries: scikit-learn^46^, matplotlib^47^, plotly^48^, and pandas^49^, and performed for the DRIFT and ATR spectral data separately. Every consecutive 10 wavenumbers were averaged to reduce the number of predictors, which decreased the computational load. Then, Principal Component Analysis (PCA) in covariance mode was implemented to observe the effect of grinding on the samples and identify potential outliers through data points far from the general cluster of points. Linear dimensionality reduction was performed using a randomized truncated singular value decomposition as proposed by Halko et al.^50^. The PCA was performed separately for ATR and DRIFT on all collected spectra. One spectral outlier was removed from the DRIFT dataset before proceeding. Hotelling’s T^2^ test was then used to determine the significance (*p* < 0.05) between the pc scores for each grinding level. Dataset was randomly split as calibration (75%) and validation (25%) sets. Partial Least Squares (PLS) regression from scikit-learn was used to build models on the calibration data for different chemical properties. The number of latent variables was used (from 1 to 30) as the tuning parameter, which was tuned using the tenfold cross-validation. The final models, which were decided based on the lowest cross validation root mean square error, were used to predict the validation set, followed by calculating statistics including R^2^ (coefficient of determination), root mean square error (RMSE), bias, and the ratio of performance to deviation (RPD), to evaluate model performances. This model calibration–validation scheme was repeated 50 times with different calibration and validation splits to estimate model uncertainties for other properties. The 50 runs of model calibration–validation yielded 50 instances of model evaluation statistics for each chemical property. An ANOVA was performed on the average model performance statistics (R^2^, RMSE, Bias, and RPD) to test the significance (*p* < 0.05) of fine grinding for each predicted property. If found to be significant, a Tukey post hoc test was performed to test the significance of these statistics between each grinding level. For the significant grinding level pairs, the mean percent change of the statistics between the two groups was calculated.

## Supplementary Information

Supplementary Information.

## Data Availability

The datasets generated during and/or analyzed during the current study are available from the corresponding author on reasonable request.
