# Supplementary material for: Effects of fine grinding on mid-infrared spectroscopic analysis of plant leaf nutrient content
Source: Sci Rep. 2023 Apr 18;13:6314. doi: 10.1038/s41598-023-33558-5 (PMC10113243; doi:10.1038/s41598-023-33558-5)
Supplement: Supplementary file 1 — Supplementary Information. [file 41598_2023_33558_MOESM1_ESM.docx]

Table S1. Particle size statistics of crop samples for each level of fine grinding.

| **Crop** | **Grind Time (min)** | **Min (µm)** | **Mean (µm)** | **Max (µm)** | **St. Dev (µm)** |
| --- | --- | --- | --- | --- | --- |
| Cereal Rye | 0 | 1.92 | 17.58 | 60.32 | 16.12 |
|  | 2 | 1.00 | 13.24 | 55.50 | 12.97 |
|  | 5 | 1.00 | 10.75 | 53.50 | 12.09 |
|  | 10 | 1.00 | 14.35 | 60.50 | 14.33 |
| Corn | 0 | 2.32 | 19.35 | 89.14 | 19.22 |
|  | 2 | 1.00 | 8.09 | 40.50 | 9.31 |
|  | 5 | 1.00 | 8.40 | 44.50 | 9.60 |
|  | 10 | 1.00 | 8.83 | 40.50 | 9.45 |
| Crimson Clover | 0 | 2.32 | 17.76 | 103.71 | 19.52 |
|  | 2 | 1.00 | 10.47 | 47.00 | 10.58 |
|  | 5 | 1.00 | 8.51 | 40.50 | 9.00 |
|  | 10 | 1.00 | 7.55 | 35.50 | 8.04 |
| Mustard | 0 | 2.50 | 25.52 | 92.46 | 20.26 |
|  | 2 | 1.00 | 11.36 | 42.00 | 9.79 |
|  | 5 | 1.00 | 7.44 | 32.00 | 7.40 |
|  | 10 | 1.00 | 9.78 | 43.50 | 9.91 |
| Rice | 0 | 2.00 | 19.95 | 85.00 | 18.20 |
|  | 2 | 1.00 | 7.24 | 32.00 | 7.20 |
|  | 5 | 1.00 | 5.73 | 26.00 | 5.67 |
|  | 10 | 1.00 | 7.23 | 32.50 | 7.56 |
| Sesame | 0 | 2.14 | 19.66 | 72.02 | 16.07 |
|  | 2 | 1.00 | 10.02 | 36.50 | 8.27 |
|  | 5 | 1.00 | 7.20 | 26.50 | 6.06 |
|  | 10 | 1.00 | 5.48 | 20.00 | 4.47 |
| Soybean | 0 | 2.00 | 16.41 | 59.50 | 13.30 |
|  | 2 | 1.00 | 10.84 | 39.50 | 8.72 |
|  | 5 | 1.00 | 8.96 | 34.50 | 7.72 |
|  | 10 | 1.00 | 6.52 | 23.50 | 5.29 |
| Triticale | 0 | 2.15 | 21.23 | 92.97 | 20.17 |
|  | 2 | 1.00 | 10.54 | 44.50 | 10.53 |
|  | 5 | 1.00 | 11.18 | 52.50 | 11.87 |
|  | 10 | 1.00 | 11.17 | 52.00 | 11.79 |
| Wheat | 0 | 1.96 | 19.62 | 85.90 | 18.64 |
|  | 2 | 1.00 | 9.26 | 38.50 | 8.89 |
|  | 5 | 1.00 | 10.30 | 53.00 | 12.13 |
|  | 10 | 1.00 | 6.34 | 30.00 | 6.51 |

Table S2. Detailed statistics of attenuated total reflectance (ATR) spectra model results.

| **Property** | **Grind Time (min)** | **Model Performance Indicator** | **Min** | **Mean** | **Max** | **St. Dev** |
| --- | --- | --- | --- | --- | --- | --- |
| Nitrogen (%) | 0 | R^2^ | 0.88 | 0.92 | 0.95 | 0.02 |
|  |  | RMSE | 0.25 | 0.31 | 0.39 | 0.03 |
|  |  | RPD | 2.80 | 3.52 | 4.57 | 0.34 |
|  |  | Bias | -0.07 | 0.00 | 0.10 | 0.04 |
|  | 2 | R^2^ | 0.87 | 0.93 | 0.95 | 0.02 |
|  |  | RMSE | 0.25 | 0.30 | 0.37 | 0.03 |
|  |  | RPD | 2.73 | 3.65 | 4.41 | 0.41 |
|  |  | Bias | -0.09 | 0.00 | 0.10 | 0.04 |
|  | 5 | R^2^ | 0.93 | 0.95 | 0.97 | 0.01 |
|  |  | RMSE | 0.19 | 0.24 | 0.29 | 0.02 |
|  |  | RPD | 3.59 | 4.60 | 6.04 | 0.55 |
|  |  | Bias | -0.08 | 0.00 | 0.06 | 0.03 |
|  | 10 | R^2^ | 0.93 | 0.95 | 0.98 | 0.01 |
|  |  | RMSE | 0.18 | 0.24 | 0.28 | 0.02 |
|  |  | RPD | 3.74 | 4.64 | 6.07 | 0.44 |
|  |  | Bias | -0.05 | 0.00 | 0.07 | 0.03 |
| Phosphorus (%) | 0 | R^2^ | 0.68 | 0.86 | 0.91 | 0.03 |
|  |  | RMSE | 0.08 | 0.09 | 0.11 | 0.01 |
|  |  | RPD | 1.53 | 2.57 | 3.27 | 0.27 |
|  |  | Bias | -0.03 | 0.00 | 0.03 | 0.01 |
|  | 2 | R^2^ | 0.71 | 0.87 | 0.93 | 0.03 |
|  |  | RMSE | 0.07 | 0.09 | 0.11 | 0.01 |
|  |  | RPD | 1.84 | 2.77 | 3.41 | 0.29 |
|  |  | Bias | -0.02 | 0.00 | 0.04 | 0.01 |
|  | 5 | R^2^ | 0.76 | 0.89 | 0.94 | 0.03 |
|  |  | RMSE | 0.07 | 0.08 | 0.11 | 0.01 |
|  |  | RPD | 1.92 | 2.89 | 3.67 | 0.37 |
|  |  | Bias | -0.02 | 0.00 | 0.03 | 0.01 |
|  | 10 | R^2^ | 0.79 | 0.91 | 0.95 | 0.03 |
|  |  | RMSE | 0.06 | 0.07 | 0.11 | 0.01 |
|  |  | RPD | 2.07 | 3.33 | 4.35 | 0.45 |
|  |  | Bias | -0.02 | 0.00 | 0.03 | 0.01 |
| Potassium (%) | 0 | R^2^ | 0.78 | 0.88 | 0.92 | 0.03 |
|  |  | RMSE | 0.43 | 0.52 | 0.70 | 0.06 |
|  |  | RPD | 2.09 | 2.81 | 3.47 | 0.29 |
|  |  | Bias | -0.14 | -0.02 | 0.13 | 0.06 |
|  | 2 | R^2^ | 0.79 | 0.88 | 0.93 | 0.02 |
|  |  | RMSE | 0.42 | 0.50 | 0.59 | 0.05 |
|  |  | RPD | 2.16 | 2.90 | 3.63 | 0.27 |
|  |  | Bias | -0.12 | -0.02 | 0.13 | 0.06 |
|  | 5 | R^2^ | 0.82 | 0.88 | 0.92 | 0.02 |
|  |  | RMSE | 0.40 | 0.52 | 0.66 | 0.06 |
|  |  | RPD | 2.27 | 2.80 | 3.50 | 0.29 |
|  |  | Bias | -0.15 | -0.01 | 0.14 | 0.07 |
|  | 10 | R^2^ | 0.83 | 0.88 | 0.94 | 0.02 |
|  |  | RMSE | 0.39 | 0.50 | 0.63 | 0.06 |
|  |  | RPD | 2.31 | 2.93 | 3.82 | 0.31 |
|  |  | Bias | -0.14 | 0.00 | 0.14 | 0.06 |
| Calcium (%) | 0 | R^2^ | 0.85 | 0.89 | 0.93 | 0.02 |
|  |  | RMSE | 0.23 | 0.29 | 0.34 | 0.02 |
|  |  | RPD | 2.45 | 2.93 | 3.60 | 0.26 |
|  |  | Bias | -0.08 | 0.01 | 0.08 | 0.035 |
|  | 2 | R^2^ | 0.82 | 0.86 | 0.92 | 0.02 |
|  |  | RMSE | 0.27 | 0.32 | 0.41 | 0.03 |
|  |  | RPD | 1.94 | 2.57 | 3.48 | 0.25 |
|  |  | Bias | -0.08 | 0.00 | 0.08 | 0.040 |
|  | 5 | R^2^ | 0.83 | 0.90 | 0.95 | 0.02 |
|  |  | RMSE | 0.20 | 0.28 | 0.37 | 0.03 |
|  |  | RPD | 2.22 | 3.05 | 4.20 | 0.37 |
|  |  | Bias | -0.06 | 0.01 | 0.07 | 0.029 |
|  | 10 | R^2^ | 0.67 | 0.84 | 0.93 | 0.06 |
|  |  | RMSE | 0.22 | 0.34 | 0.48 | 0.08 |
|  |  | RPD | 1.71 | 2.55 | 3.74 | 0.56 |
|  |  | Bias | -0.11 | 0.01 | 0.15 | 0.045 |
| Magnesium (%) | 0 | R^2^ | 0.85 | 0.89 | 0.93 | 0.02 |
|  |  | RMSE | 0.05 | 0.06 | 0.08 | 0.01 |
|  |  | RPD | 2.48 | 3.04 | 3.54 | 0.26 |
|  |  | Bias | -0.01 | 0.00 | 0.02 | 0.01 |
|  | 2 | R^2^ | 0.81 | 0.87 | 0.91 | 0.02 |
|  |  | RMSE | 0.06 | 0.07 | 0.08 | 0.00 |
|  |  | RPD | 2.30 | 2.72 | 3.23 | 0.23 |
|  |  | Bias | -0.01 | 0.00 | 0.02 | 0.01 |
|  | 5 | R^2^ | 0.78 | 0.89 | 0.92 | 0.03 |
|  |  | RMSE | 0.05 | 0.06 | 0.09 | 0.01 |
|  |  | RPD | 2.13 | 3.05 | 3.62 | 0.30 |
|  |  | Bias | -0.02 | 0.00 | 0.02 | 0.01 |
|  | 10 | R^2^ | 0.78 | 0.88 | 0.93 | 0.02 |
|  |  | RMSE | 0.05 | 0.07 | 0.09 | 0.01 |
|  |  | RPD | 2.09 | 2.78 | 3.63 | 0.30 |
|  |  | Bias | -0.02 | 0.00 | 0.03 | 0.01 |
| Iron (ppm) | 0 | R^2^ | 0.21 | 0.51 | 0.66 | 0.09 |
|  |  | RMSE | 52.1 | 77.26 | 102.25 | 12.28 |
|  |  | RPD | 0.79 | 1.32 | 1.68 | 0.22 |
|  |  | Bias | -22.92 | -0.35 | 16.52 | 6.85 |
|  | 2 | R^2^ | 0.19 | 0.47 | 0.65 | 0.09 |
|  |  | RMSE | 52.52 | 81.81 | 114.66 | 13.51 |
|  |  | RPD | 0.65 | 1.25 | 1.68 | 0.23 |
|  |  | Bias | -20.43 | -0.65 | 27.69 | 11.17 |
|  | 5 | R^2^ | 0.10 | 0.55 | 0.74 | 0.14 |
|  |  | RMSE | 55.01 | 72.26 | 106.82 | 12.32 |
|  |  | RPD | 0.74 | 1.42 | 1.92 | 0.28 |
|  |  | Bias | -16.74 | -0.76 | 22.84 | 8.81 |
|  | 10 | R^2^ | 0.40 | 0.77 | 0.91 | 0.12 |
|  |  | RMSE | 37.59 | 48.88 | 62.51 | 6.44 |
|  |  | RPD | 1.14 | 2.11 | 3.24 | 0.51 |
|  |  | Bias | -14.18 | 1.29 | 18.29 | 5.70 |
| Manganese (ppm) | 0 | R^2^ | 0.04 | 0.75 | 0.89 | 0.15 |
|  |  | RMSE | 63.71 | 83.56 | 104.67 | 9.52 |
|  |  | RPD | 0.36 | 1.91 | 2.82 | 0.54 |
|  |  | Bias | -24.68 | 1.20 | 23.55 | 10.95 |
|  | 2 | R^2^ | 0.03 | 0.70 | 0.83 | 0.15 |
|  |  | RMSE | 72.21 | 93.50 | 126.25 | 11.93 |
|  |  | RPD | 0.29 | 1.71 | 2.28 | 0.45 |
|  |  | Bias | -31.81 | 0.13 | 19.96 | 11.04 |
|  | 5 | R^2^ | 0.17 | 0.77 | 0.90 | 0.13 |
|  |  | RMSE | 59.51 | 77.59 | 95.34 | 9.33 |
|  |  | RPD | 0.40 | 2.06 | 3.05 | 0.59 |
|  |  | Bias | -18.68 | 0.93 | 25.51 | 10.06 |
|  | 10 | R^2^ | 0.13 | 0.81 | 0.91 | 0.13 |
|  |  | RMSE | 51.56 | 70.60 | 89.76 | 9.34 |
|  |  | RPD | 0.42 | 2.26 | 3.20 | 0.62 |
|  |  | Bias | -21.44 | 0.75 | 25.03 | 8.83 |
| Zinc (ppm) | 0 | R^2^ | 0.35 | 0.62 | 0.76 | 0.11 |
|  |  | RMSE | 8.16 | 10.42 | 12.85 | 1.16 |
|  |  | RPD | 1.14 | 1.60 | 2.00 | 0.21 |
|  |  | Bias | -2.46 | 0.18 | 3.18 | 1.37 |
|  | 2 | R^2^ | 0.46 | 0.72 | 0.86 | 0.09 |
|  |  | RMSE | 7.36 | 8.83 | 10.48 | 0.80 |
|  |  | RPD | 1.29 | 1.90 | 2.65 | 0.33 |
|  |  | Bias | -2.95 | -0.15 | 2.23 | 1.22 |
|  | 5 | R^2^ | 0.44 | 0.69 | 0.81 | 0.09 |
|  |  | RMSE | 7.25 | 9.39 | 11.87 | 0.92 |
|  |  | RPD | 1.24 | 1.78 | 2.26 | 0.27 |
|  |  | Bias | -1.64 | 0.04 | 3.08 | 1.17 |
|  | 10 | R^2^ | 0.46 | 0.68 | 0.82 | 0.09 |
|  |  | RMSE | 7.59 | 9.62 | 12.16 | 1.01 |
|  |  | RPD | 1.20 | 1.74 | 2.31 | 0.27 |
|  |  | Bias | -2.69 | -0.11 | 3.33 | 1.29 |
| Copper (ppm) | 0 | R^2^ | 0.68 | 0.76 | 0.86 | 0.03 |
|  |  | RMSE | 2.34 | 2.78 | 3.18 | 0.19 |
|  |  | RPD | 1.67 | 2.00 | 2.54 | 0.15 |
|  |  | Bias | -0.77 | -0.06 | 0.74 | 0.33 |
|  | 2 | R^2^ | 0.70 | 0.77 | 0.85 | 0.04 |
|  |  | RMSE | 2.26 | 2.69 | 3.19 | 0.20 |
|  |  | RPD | 1.73 | 2.07 | 2.52 | 0.18 |
|  |  | Bias | -0.82 | -0.04 | 0.55 | 0.31 |
|  | 5 | R^2^ | 0.63 | 0.78 | 0.89 | 0.04 |
|  |  | RMSE | 2.14 | 2.68 | 3.66 | 0.27 |
|  |  | RPD | 1.43 | 2.09 | 2.80 | 0.23 |
|  |  | Bias | -0.70 | -0.03 | 0.65 | 0.33 |
|  | 10 | R^2^ | 0.74 | 0.81 | 0.85 | 0.03 |
|  |  | RMSE | 2.21 | 2.48 | 2.88 | 0.15 |
|  |  | RPD | 1.84 | 2.24 | 2.57 | 0.16 |
|  |  | Bias | -0.58 | -0.05 | 0.57 | 0.31 |
| Boron (ppm) | 0 | R^2^ | 0.17 | 0.59 | 0.77 | 0.11 |
|  |  | RMSE | 19.88 | 27.02 | 40.42 | 4.30 |
|  |  | RPD | 0.67 | 1.47 | 2.05 | 0.27 |
|  |  | Bias | -6.19 | -0.20 | 6.45 | 2.96 |
|  | 2 | R^2^ | 0.14 | 0.61 | 0.84 | 0.13 |
|  |  | RMSE | 19.89 | 26.12 | 41.91 | 4.08 |
|  |  | RPD | 0.64 | 1.53 | 2.28 | 0.35 |
|  |  | Bias | -6.55 | 0.36 | 8.56 | 3.29 |
|  | 5 | R^2^ | 0.14 | 0.68 | 0.85 | 0.13 |
|  |  | RMSE | 18.75 | 23.79 | 47.25 | 4.85 |
|  |  | RPD | 0.65 | 1.69 | 2.52 | 0.37 |
|  |  | Bias | -7.92 | 0.21 | 7.37 | 3.30 |
|  | 10 | R^2^ | 0.28 | 0.60 | 0.79 | 0.12 |
|  |  | RMSE | 20.63 | 28.98 | 46.84 | 5.78 |
|  |  | RPD | 0.73 | 1.37 | 1.86 | 0.23 |
|  |  | Bias | -7.17 | 0.23 | 6.08 | 3.28 |
| Sulfur (%) | 0 | R^2^ | 0.86 | 0.91 | 0.94 | 0.02 |
|  |  | RMSE | 0.04 | 0.05 | 0.06 | 0.01 |
|  |  | RPD | 2.46 | 3.19 | 3.89 | 0.36 |
|  |  | Bias | -0.02 | 0.00 | 0.01 | 0.01 |
|  | 2 | R^2^ | 0.68 | 0.82 | 0.90 | 0.05 |
|  |  | RMSE | 0.05 | 0.07 | 0.11 | 0.01 |
|  |  | RPD | 1.30 | 2.28 | 2.79 | 0.33 |
|  |  | Bias | -0.02 | 0.00 | 0.03 | 0.01 |
|  | 5 | R^2^ | 0.71 | 0.84 | 0.91 | 0.04 |
|  |  | RMSE | 0.05 | 0.07 | 0.09 | 0.01 |
|  |  | RPD | 1.65 | 2.49 | 3.38 | 0.40 |
|  |  | Bias | -0.02 | 0.00 | 0.02 | 0.01 |
|  | 10 | R^2^ | 0.63 | 0.80 | 0.93 | 0.07 |
|  |  | RMSE | 0.04 | 0.08 | 0.11 | 0.02 |
|  |  | RPD | 1.55 | 2.25 | 3.71 | 0.53 |
|  |  | Bias | -0.02 | 0.00 | 0.02 | 0.01 |

Table S3. Detailed statistics of diffuse reflectance Fourier-transform (DRIFT) spectra model results.

| Property | Grind Time (min) | Model Performance Indicator | Min | Mean | Max | St. Dev. |
| --- | --- | --- | --- | --- | --- | --- |
| Nitrogen (%) | 0 | R^2^ | 0.85 | 0.90 | 0.94 | 0.02 |
|  |  | RMSE | 0.30 | 0.35 | 0.44 | 0.03 |
|  |  | RPD | 2.52 | 3.08 | 4.00 | 0.32 |
|  |  | Bias | -0.06 | 0.01 | 0.10 | 0.04 |
|  | 2 | R^2^ | 0.90 | 0.94 | 0.96 | 0.01 |
|  |  | RMSE | 0.22 | 0.26 | 0.31 | 0.02 |
|  |  | RPD | 3.06 | 4.16 | 5.13 | 0.45 |
|  |  | Bias | -0.06 | 0.00 | 0.07 | 0.03 |
|  | 5 | R^2^ | 0.92 | 0.94 | 0.96 | 0.01 |
|  |  | RMSE | 0.23 | 0.27 | 0.34 | 0.02 |
|  |  | RPD | 3.37 | 4.04 | 4.85 | 0.41 |
|  |  | Bias | -0.06 | 0.00 | 0.06 | 0.03 |
|  | 10 | R^2^ | 0.93 | 0.96 | 0.97 | 0.01 |
|  |  | RMSE | 0.18 | 0.23 | 0.29 | 0.02 |
|  |  | RPD | 3.82 | 4.73 | 5.97 | 0.54 |
|  |  | Bias | -0.06 | 0.00 | 0.07 | 0.03 |
| Phosphorus (%) | 0 | R^2^ | 0.61 | 0.74 | 0.84 | 0.05 |
|  |  | RMSE | 0.11 | 0.13 | 0.17 | 0.01 |
|  |  | RPD | 1.41 | 1.92 | 2.44 | 0.23 |
|  |  | Bias | -0.03 | 0.00 | 0.04 | 0.02 |
|  | 2 | R^2^ | 0.72 | 0.82 | 0.88 | 0.03 |
|  |  | RMSE | 0.09 | 0.11 | 0.14 | 0.01 |
|  |  | RPD | 1.74 | 2.30 | 2.78 | 0.24 |
|  |  | Bias | -0.04 | 0.00 | 0.02 | 0.02 |
|  | 5 | R^2^ | 0.68 | 0.81 | 0.90 | 0.04 |
|  |  | RMSE | 0.08 | 0.11 | 0.14 | 0.01 |
|  |  | RPD | 1.74 | 2.27 | 3.20 | 0.30 |
|  |  | Bias | -0.03 | 0.00 | 0.04 | 0.01 |
|  | 10 | R^2^ | 0.75 | 0.84 | 0.90 | 0.04 |
|  |  | RMSE | 0.08 | 0.10 | 0.14 | 0.01 |
|  |  | RPD | 1.81 | 2.49 | 3.07 | 0.30 |
|  |  | Bias | -0.02 | 0.00 | 0.03 | 0.01 |
| Potassium (%) | 0 | R^2^ | 0.76 | 0.86 | 0.90 | 0.03 |
|  |  | RMSE | 0.46 | 0.54 | 0.67 | 0.05 |
|  |  | RPD | 2.00 | 2.66 | 3.12 | 0.25 |
|  |  | Bias | -0.13 | 0.02 | 0.15 | 0.07 |
|  | 2 | R^2^ | 0.79 | 0.86 | 0.91 | 0.03 |
|  |  | RMSE | 0.42 | 0.55 | 0.68 | 0.05 |
|  |  | RPD | 1.96 | 2.63 | 3.28 | 0.27 |
|  |  | Bias | -0.15 | 0.02 | 0.14 | 0.06 |
|  | 5 | R^2^ | 0.79 | 0.86 | 0.92 | 0.03 |
|  |  | RMSE | 0.44 | 0.54 | 0.68 | 0.05 |
|  |  | RPD | 2.12 | 2.70 | 3.47 | 0.29 |
|  |  | Bias | -0.16 | -0.01 | 0.13 | 0.06 |
|  | 10 | R^2^ | 0.80 | 0.87 | 0.92 | 0.03 |
|  |  | RMSE | 0.44 | 0.53 | 0.73 | 0.05 |
|  |  | RPD | 2.18 | 2.74 | 3.47 | 0.27 |
|  |  | Bias | -0.16 | -0.01 | 0.12 | 0.06 |
| Calcium (%) | 0 | R^2^ | 0.85 | 0.89 | 0.94 | 0.02 |
|  |  | RMSE | 0.24 | 0.28 | 0.32 | 0.02 |
|  |  | RPD | 2.43 | 3.02 | 3.88 | 0.26 |
|  |  | Bias | -0.08 | 0.00 | 0.07 | 0.04 |
|  | 2 | R^2^ | 0.87 | 0.91 | 0.95 | 0.02 |
|  |  | RMSE | 0.21 | 0.25 | 0.29 | 0.02 |
|  |  | RPD | 2.66 | 3.37 | 4.21 | 0.33 |
|  |  | Bias | -0.07 | -0.01 | 0.06 | 0.03 |
|  | 5 | R^2^ | 0.87 | 0.92 | 0.96 | 0.01 |
|  |  | RMSE | 0.20 | 0.23 | 0.29 | 0.02 |
|  |  | RPD | 2.62 | 3.59 | 4.67 | 0.33 |
|  |  | Bias | -0.05 | 0.00 | 0.06 | 0.03 |
|  | 10 | R^2^ | 0.66 | 0.85 | 0.93 | 0.07 |
|  |  | RMSE | 0.21 | 0.33 | 0.49 | 0.08 |
|  |  | RPD | 1.69 | 2.69 | 3.62 | 0.59 |
|  |  | Bias | -0.11 | 0.01 | 0.08 | 0.04 |
| Magnesium (%) | 0 | R^2^ | 0.79 | 0.86 | 0.91 | 0.03 |
|  |  | RMSE | 0.06 | 0.07 | 0.08 | 0.01 |
|  |  | RPD | 2.07 | 2.67 | 3.32 | 0.27 |
|  |  | Bias | -0.01 | 0.00 | 0.02 | 0.01 |
|  | 2 | R^2^ | 0.82 | 0.88 | 0.92 | 0.02 |
|  |  | RMSE | 0.05 | 0.07 | 0.08 | 0.01 |
|  |  | RPD | 2.35 | 2.80 | 3.57 | 0.28 |
|  |  | Bias | -0.01 | 0.00 | 0.02 | 0.01 |
|  | 5 | R^2^ | 0.79 | 0.86 | 0.91 | 0.03 |
|  |  | RMSE | 0.06 | 0.07 | 0.09 | 0.01 |
|  |  | RPD | 2.09 | 2.65 | 3.22 | 0.28 |
|  |  | Bias | -0.02 | 0.00 | 0.02 | 0.01 |
|  | 10 | R^2^ | 0.79 | 0.88 | 0.93 | 0.03 |
|  |  | RMSE | 0.05 | 0.07 | 0.09 | 0.01 |
|  |  | RPD | 1.99 | 2.91 | 3.75 | 0.34 |
|  |  | Bias | -0.01 | 0.00 | 0.02 | 0.01 |
| Iron (ppm) | 0 | R^2^ | 0.11 | 0.31 | 0.47 | 0.08 |
|  |  | RMSE | 65.72 | 92.36 | 120.83 | 16.17 |
|  |  | RPD | 0.71 | 1.12 | 1.335 | 0.15 |
|  |  | Bias | -23.69 | -2.63 | 28.61 | 10.89 |
|  | 2 | R^2^ | 0.10 | 0.44 | 0.64 | 0.11 |
|  |  | RMSE | 57.59 | 82.75 | 114.15 | 15.66 |
|  |  | RPD | 0.67 | 1.25 | 1.65 | 0.22 |
|  |  | Bias | -15.00 | 0.08 | 23.82 | 8.80 |
|  | 5 | R^2^ | 0.08 | 0.47 | 0.64 | 0.11 |
|  |  | RMSE | 60.25 | 80.04 | 103.19 | 11.25 |
|  |  | RPD | 0.60 | 1.29 | 1.63 | 0.25 |
|  |  | Bias | -15.17 | 0.39 | 27.18 | 9.63 |
|  | 10 | R^2^ | 0.19 | 0.46 | 0.65 | 0.11 |
|  |  | RMSE | 59.31 | 80.69 | 102.53 | 11.41 |
|  |  | RPD | 0.78 | 1.27 | 1.70 | 0.24 |
|  |  | Bias | -18.73 | -0.30 | 22.35 | 8.39 |
| Manganese (ppm) | 0 | R^2^ | 0.10 | 0.56 | 0.77 | 0.14 |
|  |  | RMSE | 86.52 | 116.96 | 152.11 | 13.61 |
|  |  | RPD | 0.19 | 1.39 | 1.87 | 0.39 |
|  |  | Bias | -28.22 | 3.56 | 38.76 | 14.62 |
|  | 2 | R^2^ | 0.16 | 0.69 | 0.82 | 0.13 |
|  |  | RMSE | 68.11 | 95.49 | 140.26 | 15.02 |
|  |  | RPD | 0.34 | 1.67 | 2.29 | 0.44 |
|  |  | Bias | -29.17 | 1.38 | 33.00 | 13.31 |
|  | 5 | R^2^ | 0.05 | 0.71 | 0.87 | 0.16 |
|  |  | RMSE | 66.68 | 89.20 | 122.46 | 11.46 |
|  |  | RPD | 0.31 | 1.80 | 2.70 | 0.50 |
|  |  | Bias | -25.85 | 1.07 | 20.61 | 10.60 |
|  | 10 | R^2^ | 0.13 | 0.76 | 0.89 | 0.14 |
|  |  | RMSE | 64.11 | 82.01 | 102.65 | 8.61 |
|  |  | RPD | 0.36 | 1.95 | 2.76 | 0.55 |
|  |  | Bias | -20.83 | 1.83 | 27.82 | 10.75 |
| Zinc (ppm) | 0 | R^2^ | 0.10 | 0.45 | 0.68 | 0.11 |
|  |  | RMSE | 10.57 | 13.21 | 16.93 | 1.29 |
|  |  | RPD | 0.84 | 1.28 | 1.69 | 0.20 |
|  |  | Bias | -3.95 | 0.12 | 3.76 | 1.57 |
|  | 2 | R^2^ | 0.31 | 0.59 | 0.75 | 0.11 |
|  |  | RMSE | 8.98 | 10.84 | 14.07 | 0.98 |
|  |  | RPD | 1.08 | 1.54 | 1.92 | 0.22 |
|  |  | Bias | -3.61 | -0.15 | 3.61 | 1.27 |
|  | 5 | R^2^ | 0.15 | 0.50 | 0.71 | 0.13 |
|  |  | RMSE | 9.58 | 12.07 | 15.89 | 1.23 |
|  |  | RPD | 0.91 | 1.39 | 1.78 | 0.21 |
|  |  | Bias | -2.50 | 0.08 | 2.9 | 1.40 |
|  | 10 | R^2^ | 0.20 | 0.63 | 0.77 | 0.11 |
|  |  | RMSE | 8.09 | 10.44 | 13.99 | 1.12 |
|  |  | RPD | 0.97 | 1.60 | 2.07 | 0.24 |
|  |  | Bias | -2.53 | -0.03 | 3.34 | 1.27 |
| Copper (ppm) | 0 | R^2^ | 0.62 | 0.73 | 0.84 | 0.05 |
|  |  | RMSE | 2.49 | 2.92 | 3.49 | 0.24 |
|  |  | RPD | 1.52 | 1.89 | 2.46 | 0.19 |
|  |  | Bias | -0.88 | -0.01 | 1.26 | 0.41 |
|  | 2 | R^2^ | 0.59 | 0.68 | 0.78 | 0.04 |
|  |  | RMSE | 2.75 | 3.22 | 3.80 | 0.23 |
|  |  | RPD | 1.40 | 1.73 | 2.15 | 0.14 |
|  |  | Bias | -0.71 | -0.10 | 1.28 | 0.42 |
|  | 5 | R^2^ | 0.51 | 0.63 | 0.74 | 0.05 |
|  |  | RMSE | 3.05 | 3.44 | 3.93 | 0.22 |
|  |  | RPD | 1.38 | 1.61 | 1.89 | 0.13 |
|  |  | Bias | -0.98 | -0.11 | 1.04 | 0.49 |
|  | 10 | R^2^ | 0.65 | 0.73 | 0.80 | 0.03 |
|  |  | RMSE | 2.54 | 2.94 | 3.32 | 0.18 |
|  |  | RPD | 1.64 | 1.89 | 2.25 | 0.12 |
|  |  | Bias | -0.76 | -0.05 | 0.65 | 0.34 |
| Boron (ppm) | 0 | R^2^ | 0.07 | 0.34 | 0.52 | 0.09 |
|  |  | RMSE | 26.71 | 35.12 | 59.63 | 6.79 |
|  |  | RPD | 0.61 | 1.14 | 1.37 | 0.15 |
|  |  | Bias | -7.58 | 0.29 | 9.81 | 4.35 |
|  | 2 | R^2^ | 0.36 | 0.54 | 0.71 | 0.08 |
|  |  | RMSE | 20.40 | 28.27 | 45.85 | 4.85 |
|  |  | RPD | 0.83 | 1.40 | 1.86 | 0.21 |
|  |  | Bias | -7.62 | -0.17 | 9.55 | 3.47 |
|  | 5 | R^2^ | 0.19 | 0.51 | 0.66 | 0.09 |
|  |  | RMSE | 21.36 | 29.87 | 48.01 | 5.16 |
|  |  | RPD | 0.72 | 1.33 | 1.64 | 0.21 |
|  |  | Bias | -8.73 | 0.00 | 7.44 | 3.69 |
|  | 10 | R^2^ | 0.14 | 0.49 | 0.69 | 0.10 |
|  |  | RMSE | 21.71 | 30.46 | 46.45 | 5.42 |
|  |  | RPD | 0.76 | 1.30 | 1.62 | 0.18 |
|  |  | Bias | -6.77 | -0.32 | 6.46 | 2.78 |
| Sulfur (%) | 0 | R^2^ | 0.55 | 0.76 | 0.87 | 0.06 |
|  |  | RMSE | 0.07 | 0.08 | 0.10 | 0.01 |
|  |  | RPD | 1.35 | 2.00 | 2.70 | 0.26 |
|  |  | Bias | -0.02 | 0.00 | 0.02 | 0.01 |
|  | 2 | R^2^ | 0.57 | 0.79 | 0.89 | 0.07 |
|  |  | RMSE | 0.06 | 0.08 | 0.09 | 0.01 |
|  |  | RPD | 1.38 | 2.18 | 2.86 | 0.34 |
|  |  | Bias | -0.02 | 0.00 | 0.02 | 0.01 |
|  | 5 | R^2^ | 0.66 | 0.82 | 0.90 | 0.05 |
|  |  | RMSE | 0.05 | 0.07 | 0.08 | 0.01 |
|  |  | RPD | 1.59 | 2.34 | 3.06 | 0.35 |
|  |  | Bias | -0.02 | 0.00 | 0.01 | 0.01 |
|  | 10 | R^2^ | 0.55 | 0.74 | 0.85 | 0.07 |
|  |  | RMSE | 0.06 | 0.08 | 0.12 | 0.02 |
|  |  | RPD | 1.42 | 1.95 | 2.57 | 0.28 |
|  |  | Bias | -0.03 | 0.00 | 0.03 | 0.01 |

Table S4. Detailed statistics of properties measured from all crop types involved in this study.

| Crop Species | Number of Samples | Property | Min | Mean | Max | St. Dev. |
| --- | --- | --- | --- | --- | --- | --- |
| Amaranth | 30 | N (%) | 3.81 | 4.20 | 4.56 | 0.21 |
|  |  | P (%) | 0.45 | 0.54 | 0.68 | 0.07 |
|  |  | K (%) | 4.27 | 5.76 | 6.98 | 0.77 |
|  |  | Ca (%) | 1.62 | 1.93 | 2.31 | 0.18 |
|  |  | Mg (%) | 0.56 | 0.71 | 0.86 | 0.09 |
|  |  | S (%) | 0.15 | 0.20 | 0.24 | 0.02 |
|  |  | Cu (ppm) | 0.41 | 3.28 | 9.79 | 3.18 |
|  |  | Zn (ppm) | 18.70 | 30.82 | 44.54 | 7.02 |
|  |  | Mn (ppm) | 18.84 | 29.98 | 47.70 | 8.12 |
|  |  | Fe (ppm) | 70.69 | 102.66 | 388.11 | 61.40 |
|  |  | B (ppm) | 31.18 | 38.12 | 45.67 | 3.66 |
| Cereal Rye | 2 | N (%) | 4.53 | 4.89 | 5.26 | 0.51 |
|  |  | P (%) | 0.65 | 0.73 | 0.81 | 0.11 |
|  |  | K (%) | 4.27 | 4.30 | 4.33 | 0.04 |
|  |  | Ca (%) | 0.59 | 0.71 | 0.82 | 0.16 |
|  |  | Mg (%) | 0.23 | 0.25 | 0.26 | 0.02 |
|  |  | S (%) | 0.16 | 0.16 | 0.16 | 0.00 |
|  |  | Cu (ppm) | 5.68 | 5.71 | 5.73 | 0.04 |
|  |  | Zn (ppm) | 29.22 | 31.42 | 33.63 | 3.12 |
|  |  | Mn (ppm) | 120.36 | 129.43 | 138.50 | 12.83 |
|  |  | Fe (ppm) | 444.06 | 529.67 | 615.28 | 121.07 |
|  |  | B (ppm) | 202.74 | 213.12 | 223.50 | 14.68 |
| Corn | 78 | N (%) | 0.97 | 2.40 | 3.91 | 0.89 |
|  |  | P (%) | 0.14 | 0.34 | 0.52 | 0.08 |
|  |  | K (%) | 0.88 | 1.91 | 3.39 | 0.60 |
|  |  | Ca (%) | 0.40 | 0.93 | 1.60 | 0.30 |
|  |  | Mg (%) | 0.04 | 0.10 | 0.16 | 0.03 |
|  |  | S (%) | 0.07 | 0.12 | 0.21 | 0.03 |
|  |  | Cu (ppm) | 7.14 | 13.50 | 20.81 | 3.14 |
|  |  | Zn (ppm) | 7.69 | 23.26 | 70.27 | 10.17 |
|  |  | Mn (ppm) | 11.95 | 26.11 | 75.14 | 9.37 |
|  |  | Fe (ppm) | 56.02 | 93.91 | 238.23 | 25.98 |
|  |  | B (ppm) | 21.52 | 30.54 | 53.41 | 7.11 |
| Cotton | 30 | N (%) | 3.56 | 3.87 | 4.19 | 0.22 |
|  |  | P (%) | 0.32 | 0.49 | 0.62 | 0.08 |
|  |  | K (%) | 2.83 | 3.59 | 4.48 | 0.47 |
|  |  | Ca (%) | 2.25 | 3.04 | 3.80 | 0.34 |
|  |  | Mg (%) | 0.34 | 0.52 | 0.62 | 0.06 |
|  |  | S (%) | 0.48 | 0.67 | 0.83 | 0.11 |
|  |  | Cu (ppm) | 0.27 | 3.37 | 11.22 | 3.59 |
|  |  | Zn (ppm) | 16.22 | 21.77 | 32.91 | 4.00 |
|  |  | Mn (ppm) | 12.47 | 25.14 | 44.49 | 9.53 |
|  |  | Fe (ppm) | 64.89 | 98.37 | 145.70 | 20.53 |
|  |  | B (ppm) | 29.83 | 40.88 | 49.27 | 4.43 |
| Crimson Clover | 2 | N (%) | 4.85 | 4.88 | 4.91 | 0.04 |
|  |  | P (%) | 0.56 | 0.65 | 0.73 | 0.12 |
|  |  | K (%) | 5.88 | 6.10 | 6.32 | 0.31 |
|  |  | Ca (%) | 1.43 | 1.47 | 1.52 | 0.07 |
|  |  | Mg (%) | 0.27 | 0.30 | 0.33 | 0.04 |
|  |  | S (%) | 0.29 | 0.35 | 0.41 | 0.08 |
|  |  | Cu (ppm) | 5.49 | 5.62 | 5.75 | 0.18 |
|  |  | Zn (ppm) | 41.05 | 42.39 | 43.73 | 1.90 |
|  |  | Mn (ppm) | 87.87 | 96.95 | 106.02 | 12.83 |
|  |  | Fe (ppm) | 293.04 | 301.44 | 309.83 | 11.87 |
|  |  | B (ppm) | 387.45 | 393.66 | 399.86 | 8.78 |
| Mustard | 2 | N (%) | 5.21 | 5.36 | 5.52 | 0.22 |
|  |  | P (%) | 0.69 | 0.69 | 0.69 | 0 |
|  |  | K (%) | 5.92 | 6.43 | 6.94 | 0.72 |
|  |  | Ca (%) | 2.20 | 2.38 | 2.56 | 0.26 |
|  |  | Mg (%) | 0.30 | 0.33 | 0.36 | 0.04 |
|  |  | S (%) | 0.39 | 0.46 | 0.53 | 0.10 |
|  |  | Cu (ppm) | 5.02 | 5.30 | 5.57 | 0.39 |
|  |  | Zn (ppm) | 32.11 | 34.11 | 36.12 | 2.83 |
|  |  | Mn (ppm) | 74.21 | 75.17 | 76.12 | 1.34 |
|  |  | Fe (ppm) | 287.95 | 374.72 | 461.50 | 122.72 |
|  |  | B (ppm) | 160.05 | 161.36 | 162.66 | 1.85 |
| Rice | 15 | N (%) | 3.32 | 3.51 | 3.79 | 0.14 |
|  |  | P (%) | 0.30 | 0.48 | 0.72 | 0.15 |
|  |  | K (%) | 1.75 | 2.27 | 2.65 | 0.24 |
|  |  | Ca (%) | 0.52 | 0.62 | 0.75 | 0.07 |
|  |  | Mg (%) | 0.31 | 0.38 | 0.49 | 0.05 |
|  |  | S (%) | 0.12 | 0.15 | 0.18 | 0.01 |
|  |  | Cu (ppm) | 15.49 | 17.83 | 19.86 | 1.17 |
|  |  | Zn (ppm) | 20.39 | 24.87 | 54.59 | 8.40 |
|  |  | Mn (ppm) | 504.74 | 819.32 | 1224.9 | 210.49 |
|  |  | Fe (ppm) | 140.20 | 198.04 | 339.16 | 52.41 |
|  |  | B (ppm) | 72.10 | 94.01 | 123.37 | 16.66 |
| Sesame | 22 | N (%) | 1.63 | 3.61 | 4.72 | 0.90 |
|  |  | P (%) | 0.26 | 0.46 | 0.61 | 0.11 |
|  |  | K (%) | 1.81 | 2.29 | 3.08 | 0.40 |
|  |  | Ca (%) | 1.23 | 1.62 | 1.99 | 0.23 |
|  |  | Mg (%) | 0.24 | 0.41 | 0.49 | 0.06 |
|  |  | S (%) | 0.10 | 0.16 | 0.25 | 0.03 |
|  |  | Cu (ppm) | 5.33 | 6.59 | 8.55 | 1.09 |
|  |  | Zn (ppm) | 12.86 | 17.88 | 28.11 | 3.58 |
|  |  | Mn (ppm) | 36.31 | 51.14 | 67.81 | 7.52 |
|  |  | Fe (ppm) | 78.78 | 135.05 | 257.63 | 44.16 |
|  |  | B (ppm) | 27.80 | 37.85 | 61.96 | 9.99 |
| Sorghum | 30 | N (%) | 3.38 | 3.61 | 3.95 | 0.15 |
|  |  | P (%) | 0.39 | 0.57 | 0.84 | 0.15 |
|  |  | K (%) | 3.55 | 4.15 | 4.99 | 0.37 |
|  |  | Ca (%) | 0.36 | 0.48 | 0.70 | 0.12 |
|  |  | Mg (%) | 0.28 | 0.34 | 0.43 | 0.04 |
|  |  | S (%) | 0.14 | 0.15 | 0.18 | 0.01 |
|  |  | Cu (ppm) | 0.46 | 5.72 | 13.75 | 5.07 |
|  |  | Zn (ppm) | 24.81 | 49.44 | 98.87 | 20.58 |
|  |  | Mn (ppm) | 35.84 | 70.00 | 137.44 | 37.50 |
|  |  | Fe (ppm) | 63.27 | 77.30 | 108.18 | 11.45 |
|  |  | B (ppm) | 17.91 | 23.92 | 33.70 | 4.40 |
| Soybean | 55 | N (%) | 3.68 | 4.69 | 5.67 | 0.56 |
|  |  | P (%) | 0.40 | 0.80 | 1.89 | 0.33 |
|  |  | K (%) | 1.84 | 2.79 | 4.35 | 0.54 |
|  |  | Ca (%) | 1.24 | 1.81 | 3.45 | 0.52 |
|  |  | Mg (%) | 0.24 | 0.36 | 0.47 | 0.04 |
|  |  | S (%) | 0.08 | 0.14 | 0.25 | 0.03 |
|  |  | Cu (ppm) | 0.46 | 4.87 | 10.14 | 2.68 |
|  |  | Zn (ppm) | 18.79 | 44.53 | 130.21 | 25.94 |
|  |  | Mn (ppm) | 21.08 | 45.98 | 110.73 | 20.91 |
|  |  | Fe (ppm) | 67.75 | 152.14 | 343.75 | 75.27 |
|  |  | B (ppm) | 41.18 | 57.74 | 79.77 | 7.90 |
| Triticale | 2 | N (%) | 4.53 | 4.68 | 4.82 | 0.21 |
|  |  | P (%) | 0.72 | 0.74 | 0.75 | 0.02 |
|  |  | K (%) | 4.57 | 4.78 | 4.99 | 0.30 |
|  |  | Ca (%) | 0.55 | 0.55 | 0.56 | 0.01 |
|  |  | Mg (%) | 0.15 | 0.16 | 0.17 | 0.01 |
|  |  | S (%) | 0.13 | 0.13 | 0.13 | 0.00 |
|  |  | Cu (ppm) | 5.77 | 5.86 | 5.95 | 0.12 |
|  |  | Zn (ppm) | 29.19 | 30.86 | 32.53 | 2.36 |
|  |  | Mn (ppm) | 99.91 | 110.30 | 120.69 | 14.69 |
|  |  | Fe (ppm) | 726.21 | 899.47 | 1072.7 | 245.03 |
|  |  | B (ppm) | 211.16 | 214.64 | 218.11 | 4.92 |
| Unknown | 6 | N (%) | 3.37 | 3.52 | 3.67 | 0.16 |
|  |  | P (%) | 0.17 | 0.26 | 0.36 | 0.09 |
|  |  | K (%) | 1.19 | 3.12 | 5.15 | 2.06 |
|  |  | Ca (%) | 0.90 | 1.02 | 1.12 | 0.08 |
|  |  | Mg (%) | 0.13 | 0.19 | 0.25 | 0.06 |
|  |  | S (%) | 0.11 | 0.22 | 0.34 | 0.12 |
|  |  | Cu (ppm) | 5.26 | 5.62 | 5.96 | 0.32 |
|  |  | Zn (ppm) | 20.19 | 38.48 | 56.81 | 17.85 |
|  |  | Mn (ppm) | 29.32 | 55.01 | 81.78 | 25.66 |
|  |  | Fe (ppm) | 73.80 | 83.64 | 91.82 | 6.61 |
|  |  | B (ppm) | 28.21 | 31.12 | 34.25 | 2.37 |
| Wheat | 32 | N (%) | 4.32 | 5.04 | 5.36 | 0.19 |
|  |  | P (%) | 0.73 | 0.85 | 1.01 | 0.08 |
|  |  | K (%) | 3.38 | 4.72 | 5.41 | 0.61 |
|  |  | Ca (%) | 0.35 | 0.41 | 0.52 | 0.05 |
|  |  | Mg (%) | 0.19 | 0.22 | 0.25 | 0.01 |
|  |  | S (%) | 0.15 | 0.20 | 0.22 | 0.02 |
|  |  | Cu (ppm) | 0.46 | 8.05 | 16.85 | 4.97 |
|  |  | Zn (ppm) | 21.90 | 31.11 | 47.72 | 5.92 |
|  |  | Mn (ppm) | 23.39 | 58.61 | 121.27 | 33.06 |
|  |  | Fe (ppm) | 75.19 | 132.57 | 914.74 | 155.65 |
|  |  | B (ppm) | 35.89 | 55.17 | 256.66 | 49.46 |
